# The lipopeptides pseudofactin II and surfactin effectively decrease *Candida albicans* adhesion and hydrophobicity

**DOI:** 10.1007/s10482-015-0486-3

**Published:** 2015-05-29

**Authors:** Piotr Biniarz, Gabriela Baranowska, Joanna Feder-Kubis, Anna Krasowska

**Affiliations:** Faculty of Biotechnology, University of Wrocław, ul. Fryderyka Joliot-Curie 14a, 50-383 Wrocław, Poland; Faculty of Chemistry, Wrocław University of Technology, Wybrzeże Wyspiańskiego 27, 50-370 Wrocław, Poland

**Keywords:** *Candida albicans*, Lipopeptides, Biosurfactants, Adhesion, CSH

## Abstract

A serious problem for humans is the propensity of *Candida albicans* to adhere to various surfaces and its ability to form biofilms. Surfactants or biosurfactants can affect the cell surfaces of microorganisms and block their adhesion to different substrates. This study investigated adhesion of *C. albicans* strains differing in cell surface hydrophobicity (CSH) to polystyrene microplates in order to compare the ability of lipopeptide biosurfactants pseudofactin (PF II) and surfactin (SU) to prevent fungal adhesion to polystyrene. The biosurfactants decreased adhesion of tested strains by 35–90 % when microplates were conditioned before the addition of cells. A 80–90 % reduction of adhesion was observed when cells were incubated together with lipopeptides in microplates. When microplates were pre-coated with biosurfactants, PF II was less active than SU, but when cells were incubated together with biosurfactants, the activity of both compounds was similar, independent of the CSH of strains. When cells were preincubated with lipopeptides and then the compounds were washed out, the adhesion of hydrophobic strains increased two times in comparison to control samples. This suggests irreversible changes in the cell wall after the treatment with biosurfactants. CSH of hydrophobic strains decreased only by 20–60 % after incubation with biosurfactants while adhesion decreased by 80–90 %; the changes in cell adhesion can be thus only partially explained through the modification of CSH. Preincubation of *C. albicans* with biosurfactants caused extraction of cell wall proteins with molecular mass in the range of 10–40 kDa, which is one possible mechanism of action of the tested lipopeptides.

**Electronic supplementary material:**

The online version of this article (doi:10.1007/s10482-015-0486-3) contains supplementary material, which is available to authorized users.

## Introduction

*Candida albicans* is responsible for fungaemia, especially in immunocompromised patients. Cell features that cause mycoses encompass, e.g., adhesion, secretion of hydrolytic enzymes, filamentation and hydrophobicity (Verstrepen and Klis 2006). Understanding how *C. albicans* morphogenesis modulates the molecular composition of the fungal cell surface and interactions with biotic and abiotic surfaces is important, but still unclear.

The microbial adhesion results from specific interactions between cell surface structures and the surface of the substrate, or from non-specific interaction forces, including Brownian movement, van der Waals attraction, gravitational forces and surface electrostatic charges. One of the important factors is the hydrophobicity of cell surface (Krasowska and Sigler 2014).

Cell surface hydrophobicity (CSH) is connected with adhesion and pathogenic processes of *C. albicans*. Hydrophobic cells are more adherent than hydrophilic ones to epithelial and endothelial tissues as well as to abiotic surfaces (Glee et al. 2001; Hazen 2004).

Hydrophobicity of *C. albicans* cells alters in response to changes in environmental conditions (e.g. temperature, composition of medium) and growth phases (Hazen et al. 2001) and can be switched between hydrophilic and hydrophobic phenotypes (Masuoka and Hazen 1997). Hydrophilic cells have an elongated acid-labile mannan fraction in the cell wall and the length of this structure affects the folding of cell wall fibrils (Masuoka and Hazen 1999).

Chaffin (2008) supposed that Csh1 protein influences the acid-labile mannan composition, because of differences between hydrophobic and hydrophilic cells in mannan fractions. Mannoproteins can therefore be potential targets for new antifungal drugs (Gow et al. 1999).

Biosurfactants such as lipopeptides are particularly interesting as antifungals because of their high surface activity and antibiotic potential. Several natural lipopeptides, e.g. echinocandins, block specific enzymatic reactions in the synthesis of cell wall components (e.g. β-1,3-glucan or chitin). Lipopeptides such as surfacin (SU), iturin and bafilomycin disturb the plasma membrane (Makovitzki et al. 2006). The adsorption of biosurfactant molecules on a surface was found to change its hydrophobicity, which might cause changes in the adhesion processes (Zhong et al. 2008; Singh et al. 2013).

Previously, we described the antiadhesive activity of the lipopeptide pseudofactin II (PF II), produced by *Pseudomonas fluorescens* BD5 (Janek et al. 2010) against several uropathogenic bacteria and *C. albicans*, and did not detect a significant impact on *C. albicans* growth (Janek et al. 2012).

PF II and SU are both cyclic lipopeptides. In the PF II molecule a palmitic acid is connected to hydrophilic “head” of eight uncharged amino acids (Janek et al. 2010), whereas SU is a lipoheptapeptide linked to a β-hydroxyl fatty acid. Commercially available SU (Sigma-Aldrich) is a mixture of congeners that differ in the length of the carbon chain (C_12_–C_16_). Moreover SU is negatively-charged because of Asp and Glu amino acids within the molecule (Raaijmakers et al. 2010). These differences cause a variations in the biological activity of lipopeptides e.g. disruption of plasma membrane by SU.

In this work we compared the action of PF II and SU on *C. albicans* strains that differ in CSH. We examined the influence of lipopeptides on the viability and adhesion of *C. albicans* on polystyrene. We also checked the impact of the biosurfactants on CSH of *C. albicans*. Our results suggest differences in the mechanisms of action between PF II and SU. Micelles of PF II and SU cause irreversible changes in the cell wall of hydrophobic strains of *C. albicans* but a decrease in adhesion could be explained only partially by the influence of lipopeptides on CSH. Moreover, the biosurfactants appeared to be able to extract some cell surface-associated proteins from *C. albicans* cell wall (CWP), which is demonstrated for the first time in this work.

## Materials and methods

### Microorganisms and culture conditions

Biosurfactant-producing strain *P. fluorescens* BD5, obtained from freshwater from Arctic Archipelago of Svalbard, was cultivated in LB medium as described earlier (Janek et al. 2010). *C. albicans* strains (Table 1) were a generous gift from D. Sanglard (Lausanne, Switzerland) and were cultivated in 5 ml YPG broth containing 10 g/l bactopeptone (Difco, USA), 10 g/l yeast extract (Difco, USA), and 20 g/l glucose (Bioshop, Canada). *Candida* cultures were incubated at 28 °C for 24 h without agitation and then stored at 4 °C for a maximum of 2 weeks. All experiments were carried out on fresh *C. albicans* pre-cultures (4.85 ml of YPG inoculated with 150 µl of *C. albicans* culture and incubated for 24 h at 28 °C). Before conducting the experiments *C. albicans* cells were centrifuged twice (1000×*g*) for washing out the culturing medium and resuspended in PBS pH = 7.4 (8 g/l NaCl, 1.4 g/l Na_2_HPO_4_, 0.25 g/l KH_2_PO_4_, 0.2 g/l KCl) or phosphate buffer (PB; 16.9 g/l K_2_HPO_4_, 7.3 g/l KH_2_PO_4_).

**Table 1:** *Candida albicans* strains used in work

Strain	Genotype	Reference
SC5314	Clinical isolate	(Gillum et al. 1984)
CAF2-1	Δura3::imm434/URA3	(Fonzi and Irwin 1993)
CAF4-2	Δura3::imm434/Δura3::imm434	(Fonzi and Irwin 1993)
DSY653	Δcdr2::hisG/Δcdr2::hisG-URA3-hisG	(Sanglard et al. 1997)
DSY1050	Δcdr1::hisG/Δcdr1::hisG Δcdr2::hisG/Δcdr2::hisG Δmdr1::hisG-URA3-hisG/Δmdr1::hisG	(Mukherjee and Chandra 2003)

### Production, isolation and purification of pseudofactin II (PFII)

For the production of PFII, *P. fluorescens* BD5 was cultivated in mineral salt medium (MSM) containing 7 g/l K_2_HPO_4_, 2 g/l KH_2_PO_4_, 1 g/l (NH_4_)_2_SO_4_, 0.5 g/l sodium citrate × 2H_2_O, and 0.1 g/l MgSO_4_ × 7H_2_O supplemented with 20 g/l glucose at 28 °C without agitation as described earlier (Janek et al. 2010). Briefly, 0.5 l of MSM was inoculated with 5 ml of *P. fluorescens* BD5 culture in LB (24 h, 28 °C) and incubated for 1 week at 28 °C without agitation. Cell-free supernatant was afterwards extracted three times with ethyl acetate. The solvent was evaporated under vacuum and crude extract was dissolved in methanol and purified by RP-HPLC (Janek et al. 2010).

### Biosurfactant concentrations

Biosurfactants were tested in the final concentrations of 0.035 or 0.1 mg/ml for PFII and 0.005 or 0.015 mg/ml for SU. These concentrations were chosen to test the influence of biosurfactant monomers (~0.5 × CMC) and micelles (~1.5 × CMC). PFII was extracted and purified as described above. SU was manufactured by Sigma-Aldrich (USA). Biosurfactant stock solutions were dissolved in PBS and stored at −20 °C.

### Antifungal activity of biosurfactants

The antifungal activity of biosurfactants was tested in 96-well flat-bottom polystyrene microplates (Sarstedt, Germany). We added 50 µl of double strength YPG and 50 µl of biosurfactant solution in PBS to each well or PBS to control wells. Every well was afterwards inoculated with overnight *Candida* culture in YPG to reach the initial optical density at 600 nm (OD_600_) of 0.01. The microplates were then incubated for 24 h at 30 °C. After incubation the OD_600_ was measured with UMV 340 microplate reader (Asys Hitech, Austria). Antifungal activity of biosurfactants is expressed as a growth inhibition in comparison to samples without biosurfactants (100 %):

$$ Growth\,inhibition \left( \% \right) = 100 \times \left[ {1 - \frac{{OD_{T} }}{{OD_{C} }}} \right] $$

where OD_T_ is the OD_600_ of wells containing biosurfactants in PBS and OD_C_ is the OD_600_ of control samples (wells without biosurfactants).

### Cell surface hydrophobicity (CSH)

For determining the effect of biosurfactants on *C. albicans* CSH, cell suspensions in PB were transferred to Eppendorf test tubes and PFII or SU stock solutions in PBS were added to reach the biosurfactant final concentrations. The same amount of PBS was added to the control samples. Suspensions were incubated for 2 h at 37 °C with agitation (300 rpm) and then diluted to an OD_600_ of 0.5. The MATH (microbial adhesion to hydrocarbon) was used to evaluate the CSH of *Candida* cells (Coimbra et al. 2009). Briefly, 2 ml of the cell suspension in PB were moved to a glass tube (100 × 15.5 mm) and 100 µl of hexadecane w added. The samples were then vortex-shaken for 3 min and the phases were allowed to separate for 1 h. The OD_600_ of the aqueous phase was measured and CSH, defined as percentage of cells adhering to hexadecane, was calculated as follows:

$$ CSH \left( \% \right) = 100 \times \left[ {1 - \frac{{OD_{600} }}{0.5}} \right] $$

where OD_600_ is the optical density of the aqueous phase at 600 nm. In modified trials, biosurfactants were washed out (centrifugation 1000×*g*) with PB before diluting cell suspensions to an OD of 0.5 and measuring CSH.

### Adhesion of *Candida albicans* to polystyrene

PF II and SU were tested as *C. albicans* adhesion-inhibiting agents in flat-bottom 96-well polystyrene microplates (Sarstedt, Germany) in three different assays. In pre-adhesion assay, microplate wells were preincubated with 100 µl of biosurfactant solutions in PBS for 2 h at 37 °C with agitation (300 rpm). PBS buffer was used as a positive control. Subsequently, wells were washed two times with PBS. *C. albicans* suspensions in PBS were diluted to give an OD_600_ of 0.6. The highest adhesion of *C. albicans* strains to polystyrene was observed at this OD (Janek et al. 2012). 100 µl of *Candida* suspensions were added to wells and incubated for 2 h at 37 °C with agitation (300 rpm). Then supernatants were removed and wells were washed two times with PBS to remove non-adherent cells. Adherent cells were stained with 0.1 % crystal violet for 5 min and then wells were washed three times with PBS. The dye was released by 200 µl of 0.05 M HCl with 1 % SDS in isopropanol and the absorbance at 590 nm (Abs_590_) was read off with Asys UMV 340 microplate reader (Asys Hitech, Austria). Cell adhesion was expressed as the Abs_590_ or as the percentage of Abs_590_ of control samples (100 %):

$$ Adhesion \left( \% \right) = 100 \times \left[ {1 - \frac{{Abs_{T} }}{{Abs_{C} }}} \right] $$

where Abs_t_ is the Abs_590_ of wells pretreated with biosurfactants and Abs_c_ is the Abs_590_ of control wells (pretreated with PBS only). In addition, we tested *C. albicans* adhesion to microplates in the presence of biosurfactants. Briefly, we added biosurfactants to *Candida* suspensions in PBS to reach final concentrations and the OD_600_ of 0.6. The same amount of PBS was added to the control samples. Then, 100 µl of suspensions were added to microplate wells and incubated for 2 h at 37 °C with agitation (300 rpm). The microplates were washed, stained and read as described before. We also investigated the influence of preincubation of *C. albicans* strains with biosurfactants on their adhesion abilities. In brief, *Candida* cell suspensions in PBS were transferred to Eppendorf test tubes and biosurfactants were added to the desired final concentrations. The same amount of PBS was added to the control samples. Suspensions were incubated for 2 h at 37 °C with agitation (300 rpm) and diluted to an OD_600_ of 0.6. Then, 100 µl of suspensions were added to microplate wells and incubated for 2 h at 37 °C with agitation (300 rpm). Microplates were washed, stained and read as described before. In modified trials, biosurfactants were washed out (centrifugation 1000×*g*) with PBS before diluting cell suspensions to an OD_600_ of 0.6 and conducting the adhesion assay.

### Extraction of cell-wall associated proteins (CWP) by biosurfactants

We also tested if the addition of biosurfactants can cause extraction of proteins from the *C. albicans* cell surface. To conduct the experiment, *Candida* cell suspensions in PBS were transferred to Eppendorf test tubes and biosurfactants were added to the final concentrations. The same amount of PBS was added to the control samples. Suspensions were incubated for 2 h at 37 °C with agitation (300 rpm). Then cells were removed by centrifugation (1000×*g*) and filtration (0.2 µm). Proteins in supernatants were concentrated with Amicon Ultra 0.5 mL 3 kDa centrifugal filters (Millipore, USA). Concentrated samples were mixed with ×6 denaturation buffer (150 mM Tris; 0.6 M EDTA; 12 % SDS; 60 mM DTT), heated at 95 °C for 5 min and loaded onto 15 % polyacrylamide gel. Silver-stained gels were photographed with ChemiDoc System (Bio-Rad, USA).

### Fluorescence microscopy

*Candida* cell suspensions in PBS were transferred to Eppendorf test tubes and biosurfactants were added to the final concentrations. The same amount of PBS was added to the control samples. SDS was added to the final concentration of 1 % and served as positive-control samples. Suspensions were incubated for 2 h at 37 °C with agitation (300 rpm) as described above. Then cells were centrifuged twice (1000×*g*) and resuspended in PBS buffer. PI from stock solution (Bioshop, Canada) was added to the final concentration of 6 µM and suspensions were incubated for 5 min at room temperature. Next, *Candida* cells were pelleted and washed twice with PBS. 4 µl of *Candida* pellets were viewed with Zeiss Axio Imager A2 fluorescence microscope.

### Statistical analysis

All described assays were carried out at least three times in three replicates. Statistical analyses were performed using paired *t* test with Bonferroni correction. *P* values of <0.05 were considered significant.

## Results and discussion

*C. albicans* can use various carbon sources: glucose, galactose, fructose or hydrocarbons. Carbon sources at different concentrations promote changes in the structure of cell wall (McCourtie and Douglas 1981); thus increasing sugar concentration in the medium from 50 to 500 mM resulted in the production of an outer fibrillar-floccular layer of mannoproteins and also a linear increase of adherence to acrylic surfaces (McCourtie and Douglas 1981). Different culture conditions have therefore an impact on surface properties of *Candida* cells (Hobden et al. 1995).

**Fig. 1:**
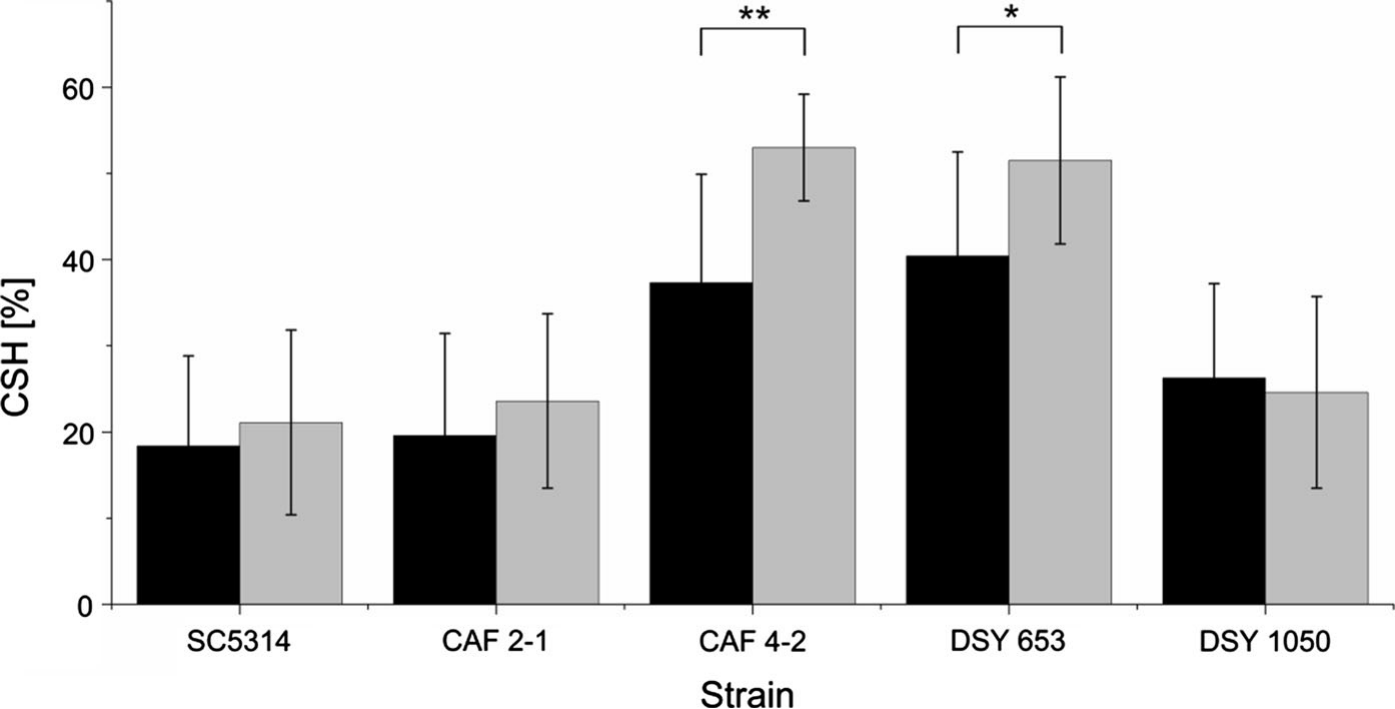
Cell surface hydrophobicity (CSH) of *C. albicans* strains cultivated in YPG medium supplemented with 0.2 % glucose (*black bars*) or 2 % glucose (*grey bars*). Differences in CSH between strains cultivated in different media were analyzed using modified paired Student *t* test *P < 0.05, **P < 0.001

In our collection of *C. albicans* strains (Table 1), CAF4-2 and DSY653 were more hydrophobic than other strains (*P* < 0.001) (Fig. 1).

A change in glucose concentration in the medium from 2 to 0.2 % decreased CSH but only in the case of two strains with the highest hydrophobicity (Fig. 1). These results suggest differences in cell wall composition and metabolism of URA3 mutants as reported earlier (Bain et al. 2001). Our results also indicate an impact of the site of integration of URA3 in *C. albicans* genome on changes in surface properties. Strains DSY653 and DSY1050 that vary in the site of integration of URA3 differ in some aspects such as CSH (Fig. 1).

**Fig. 2:**
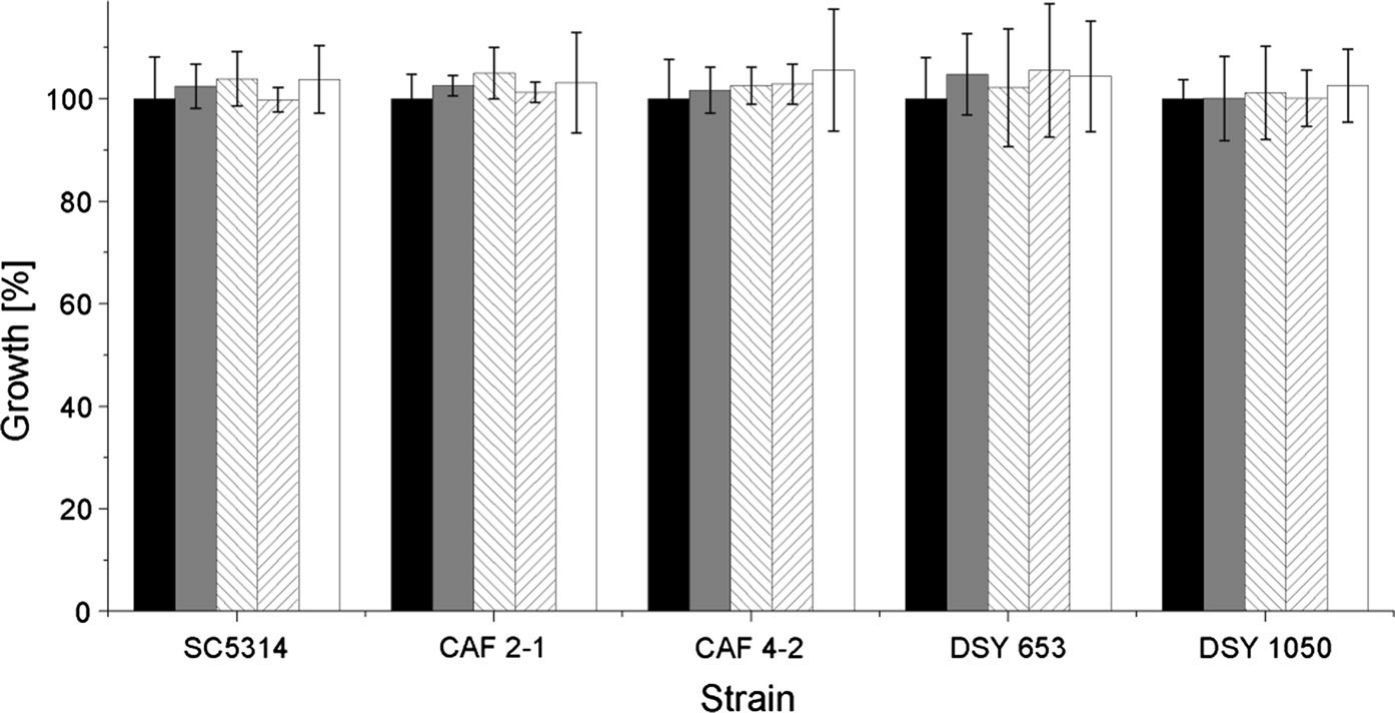
Growth of *C. albicans* strains in the presence of 0.035 mg/ml (*grey bars*) and 0.1 mg/ml (*inverse-hatched bars*) PF II in PBS or 0.005 mg/ml (*hatched bars*) and 0.015 mg/ml (*white bars*) SU in PBS, compared to control (*black bars*)

Microbial surfactants often have antimicrobial properties but knowledge about mechanisms of their action is scarce. A few studies have shown that rhamnolipids increase the membrane permeability and alter its barrier function, causing cell damage (Sotirova et al. 2008). Lipopeptides such as SU, iturin or lichenisyn form ion-conducting membrane channels (Pueyo et al. 2009; Bensaci et al. 2011). In contrast to many other lipopeptides (Peypoux et al. 1999; Grangemard et al. 2001), PF II showed much weaker antimicrobial activity against bacterial and *C. albicans* strains (Janek et al. 2012). Also SU in tested concentrations exhibited no antifungal activity (Fig. 2). PF II was found to possess an antiadhesive, concentration-dependent activity against bacteria and yeast. The highest reduction of adhesion (80–99 %) was observed for *C. albicans* wild-type strain SC5314 (Janek et al. 2012). PF II was effective above the critical micelle concentration (0.072 mg/ml) and the adhesion was thus inhibited more strongly by micelles than by monomers (Janek et al. 2012). The microbial adhesion depends on the composition of the outer cell layer and is connected with hydrophobic/hydrophilic and ionic properties of the cell as well as with the properties of the polystyrene surface of microplates used in experiments (Neu 1996). PF II, due to its nonionic character, can probably coat positively or negatively charged surfaces, changing their properties.

We studied the adhesion of *C. albicans* to polystyrene microplates in a number of different experiments to compare the ability of PF II and SU to prevent fungal adhesion to abiotic surfaces. It is obvious that strains CAF4-2 and DSY653 have modified surface properties, but the nature of these changes is not clear (Bain et al. 2001).

**Fig. 3:**
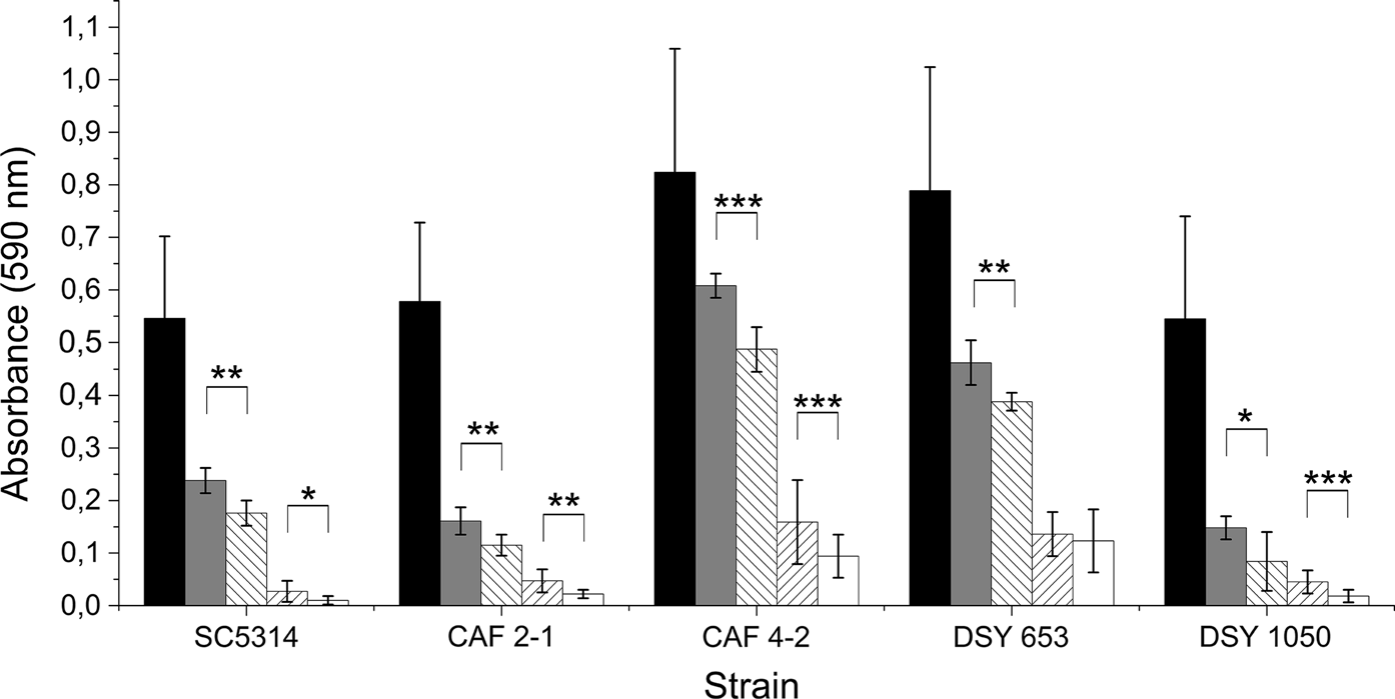
Adhesion of *C. albicans* strains to polystyrene microplates pretreated with 0.035 mg/ml (*grey bars*) and 0.1 mg/ml (*inverse-hatched bars*) PF II in PBS or 0.005 mg/ml (*hatched bars*) and 0.015 mg/ml (*white bars*) SU in PBS, compared to control adhesion (*black bars*). Statistical analysis was performed with modified paired *t* test *P < 0.05, **P < 0.01, ***P < 0.001

We observed a decrease in adhesion of all tested *C. albicans* strains when the microplates were pretreated with PF II before the addition of the microorganisms (pre-adhesion assay) (Fig. 3). PF II was more active in concentrations higher than CMC (0.1 mg/ml) (Fig. 3). We observed a similar concentration-dependent effect for SU used as a standard lipopeptide biosurfactant, which decreased the adhesion even more than PF II (*P* < 0.001) (Fig. 3). CAF4-2 and DSY653 adhered to the polystyrene microplate surface better than the other strains (*P* < 0.01) and were able to adhere to a surface pretreated with lipopeptides more strongly than other strains (*P* < 0.001) (Fig. 3).

**Fig. 4:**
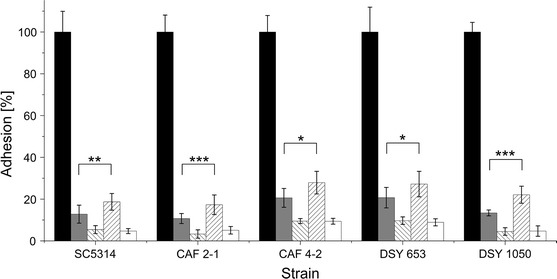
Adhesion of *C. albicans* strains in the presence of 0.035 mg/ml (*grey bars*) and 0.1 mg/ml (*inverse hatched bars*) PF II in PBS or 0.005 mg/ml (*hatched bars*) and 0.015 mg/ml (*white bars*) SU in PBS, compared to adhesion of strains incubated in PBS (*black bars*) after 2-h incubation in 37 °C. Statistical analysis was performed with a modified paired *t* test *P < 0.05, **P < 0.01, ***P < 0.001

Surprisingly, when cells and lipopeptides were incubated together for 2 h in the polystyrene microplate, the adhesion was blocked even more strongly (Fig. 4). Both PF II and SU micelles reduced *C. albicans* adhesion by ~90 %. As for biosurfactant monomers, the action of lipopeptides was different. In this case, PF II was found to be a better antiadhesive agent than SU (Fig. 4). The antiadhesive activity of SU was similar to the situation when it coated the microplate before the addition of *Candida* suspension (cf. Figs. 3, 4). PF II was less active than SU in the case of hydrophobic strains when the microplate was coated before the addition of cells but when hydrophobic cells were incubated together with PF II, their adhesion decreased like in hydrophilic strains (Figs. 3, 4). These results suggest differences in the mechanisms of action between PF II and SU, e.g. interactions between cell surface and/or polystyrene.

**Fig. 5:**
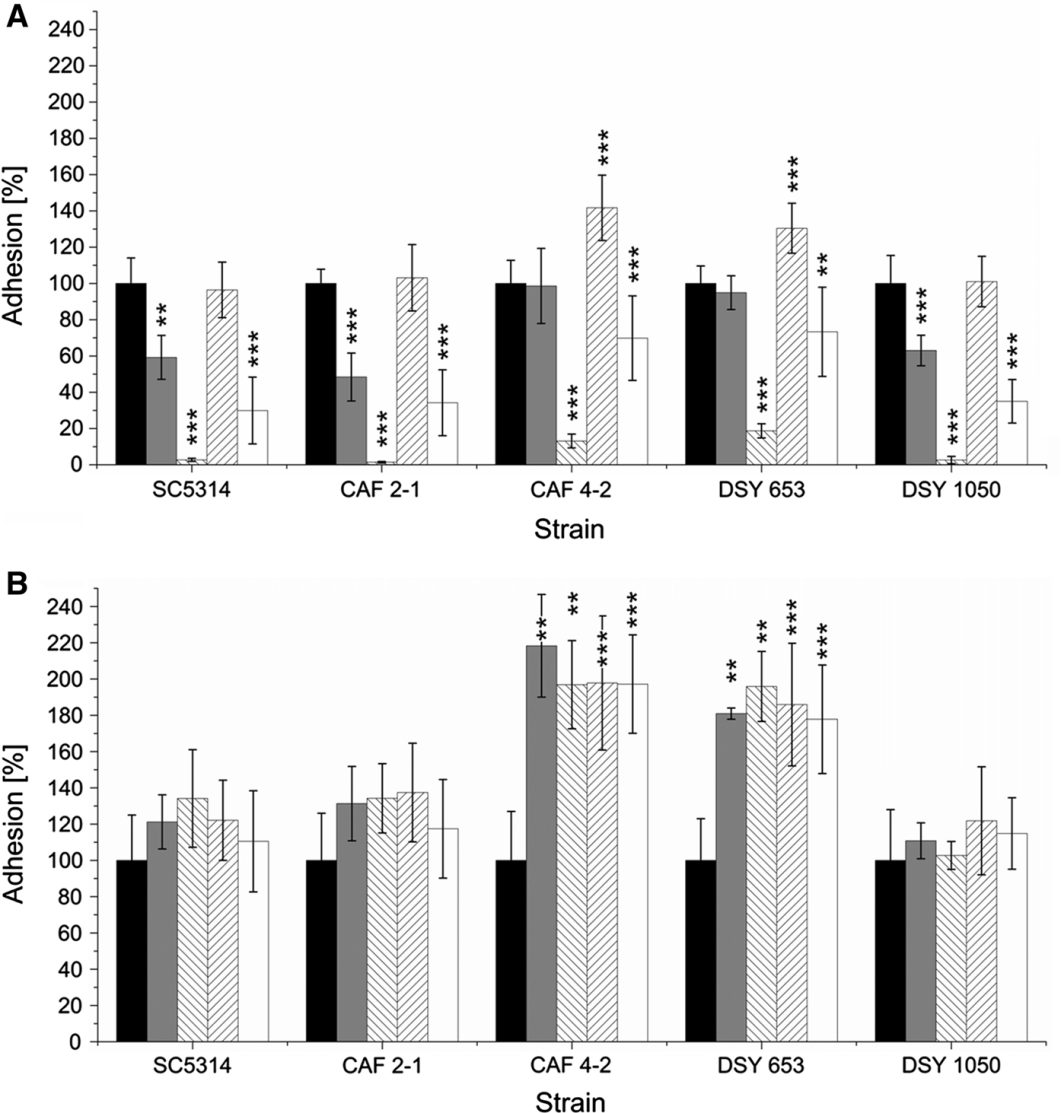
Adhesion of *C. albicans* cells preincubated with 0.035 mg/ml (*grey bars*) and 0.1 mg/ml (*inverse hatched bars*) PF II in PBS or 0.005 mg/ml (*hatched bars*) and 0.015 mg/ml (*white bars*) SU in PBS, compared to adhesion of strains preincubated in PBS (*black bars*). Two different assays were performed: biosurfactants were present in solution during adhesion test (**a**) or were washed out after 2 h of preincubation prior to adhesion test (**b**). Statistical analysis was performed by modified paired *t* test * *P* < 0.05, ** *P* < 0.01, *** *P* < 0.001

Interesting results were observed when the cells were preincubated with biosurfactants and the adhesion of coated and non-coated cells to polystyrene microplate was investigated (Fig. 5). When present in the solution (Fig. 5a), lipopeptides act as strong antiadhesives in micellar concentrations. PF II monomers reduced the adhesion of hydrophilic strains approximately two times and did not alter adhesion of hydrophobic strains CAF4-2 and DSY653 (Fig. 5a). Monomers of SU did not change adhesion of hydrophilic strains and increased it in the case of hydrophobic strains (Fig. 5a). During incubation of *Candida* cells with the biosurfactants, the predisposition of cells to adhesion changed and was different from the case when the microplate was pre-coated with PF II or SU (Figs. 3, 5). However, micelles of PF II decreased adhesion to the same low level (10–20 %) as in experiments with a 2-h adhesion of cells coated with PF II (Fig. 4). When the biosurfactants were washed out before conducting the experiment, the adhesion of hydrophilic strains was comparable to control samples whereas for hydrophobic strains adhesion increased approximately two times (Fig. 5b). This result suggests irreversible changes in the cell wall of hydrophobic strains of *C. albicans* caused by micelles of PF II and SU after a 2-h incubation.

The microbial ability of adhering to different surfaces is connected with CSH, hence our intention was to investigate the influence of lipopeptides on *Candida* CSH. Biosurfactants can change CSH due to adsorbing to the cell surface (Kaczorek et al. 2013), like rhamnolipids which strongly adsorbed on the cell surface of yeast (Kaczorek et al. 2008).

**Fig. 6:**
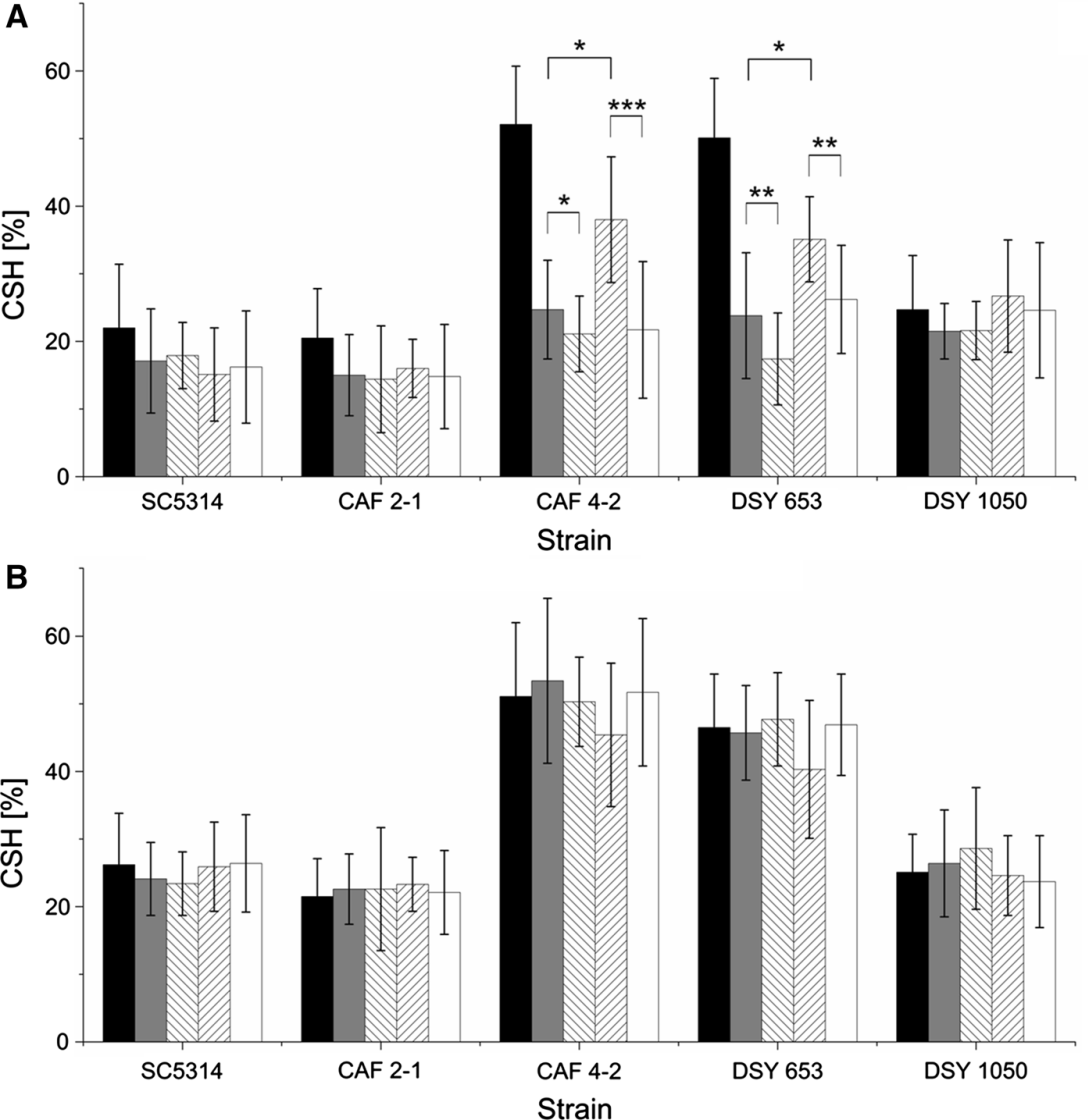
Cell surface hydrophobicity (CSH) of *C. albicans* strains pretreated with 0.035 mg/ml (*grey bars*) and 0.1 mg/ml (*inverse hatched bars*) PF II in PBS or 0.005 mg/ml (*hatched bars*) and 0.015 mg/ml (*white bars*) SU in PBS, compared to control samples (*black bars*). The CSH was measured in the presence of biosurfactants in *Candida* suspension (**a**) or after rinsing out the surface-active compounds (**b**). Statistical analysis was performed by modified paired *t* test *P < 0.05, **P < 0.01, ***P < 0.001

After a 2-h incubation with PF II or SU, CSH of *C. albicans* CAF4-2 and DSY653 significantly decreased and this effect was concentration-dependent. Monomers of PF II influenced CAF4-2 and DSY653 more strongly than monomers of SU. Other tested strains seemed resistant to the influence of biosurfactants (Fig. 6a). On the other hand, when biosurfactants were washed out, CSH level of hydrophobic cells recovered (Fig. 6b). In this assay the time of incubation with biosurfactants was 2 h and these conditions can be compared to experiments with adhesion of cells treated with biosurfactants (Fig. 4). CSH of hydrophobic strains decreased only by 20–60 % (Fig. 6) while adhesion decreased by 80–90 % (Fig. 4). Also the potential irreversible changes in the cell surface of *C. albicans* caused by lipopeptides have an impact on adhesion but not on CSH of hydrophobic strains (cf. Figs. 5, 6). This result suggests that decrease in cell adhesion by lipopeptides can be only partially explained by the modification of CSH and should be considered only in the case of hydrophobic strains CAF4-2 and DSY653.

**Fig. 7:**
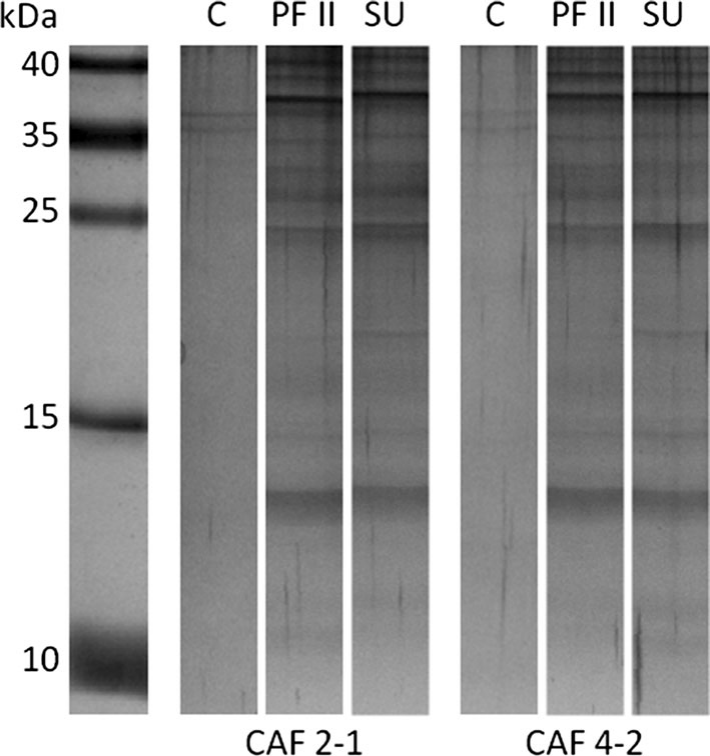
SDS-PAGE electrophoresis of proteins obtained after incubation of *C. albicans* cells with 0.1 mg/ml PF II or 0.015 mg/ml SU in comparison to control samples (C)

One of the mechanisms of action of lipopeptides on *C. albicans* cells could be a decrease in the level of some compounds (e.g. chitin, β-1,3-glucan) in the cell wall (Bizerra et al. 2011). Some protocols for the fractionation of fungal cell walls include treatment with synthetic surfactants (Pitarch et al. 2002; Klis et al. 2007). Therefore, we isolated several proteins from cell-free supernatants after preincubation of *C. albicans* cells with biosurfactants and visualized them on silver-stained polyacrylamide gels (Fig. 7). We determined molecular masses of these proteins after SDS-PAGE electrophoresis to be in the range from ~10 to 40 kDa and observed no differences between the action of PF II and SU or between hydrophobic and hydrophilic strains (Fig. 7). Simultaneously, PAS (Periodic acid-Schiff) staining for glycoproteins showed no bands on the gels (data not shown). Therefore, partial disruption of cell wall and extraction of cell surface-associated proteins can be the possible mechanism of the action of lipopeptide biosurfactants on *C. albicans*.

**Fig. 8:**
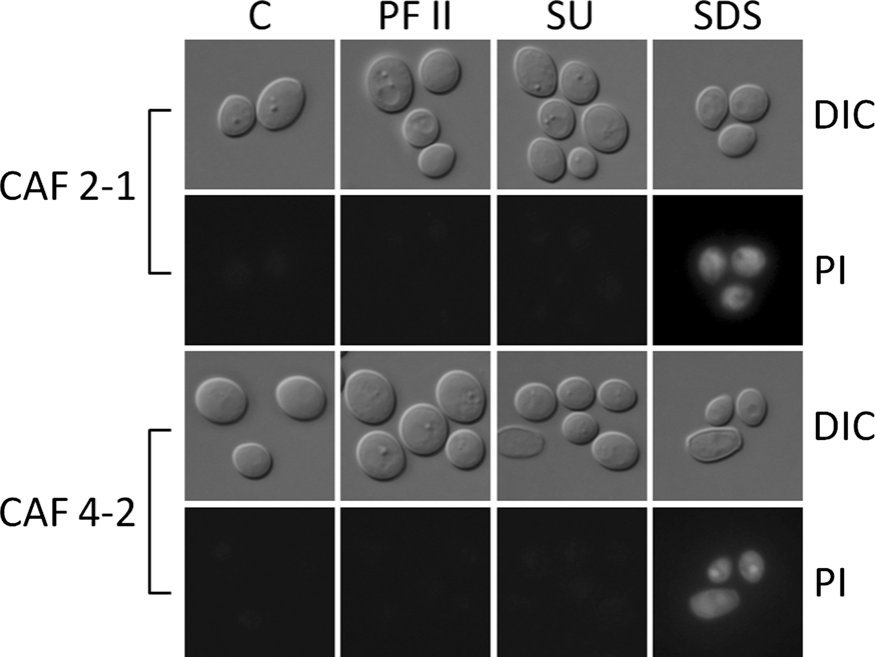
Propidium iodine (PI) fluorescence and differential interference contrast (DIC) microphotographs of *C. albicans* cells incubated with 0.1 mg/ml PF II or 0.015 mg/ml SU in comparison with control samples (C) and 1 % SDS as positive controls

To exclude the possibility of contamination of cell-free supernatants (Fig. 7) with cytoplasmic proteins, we analyzed viability and membrane permeability of *Candida* cells with fluorescence microscopy (Fig. 8). The lack of propidium iodine (PI) fluorescence in control samples and cells incubated with lipopeptides indicate that cells were viable and membranes permeability was undisturbed (Fig. 8), which also confirms viability results shown earlier (Fig. 2). In contrast, cells treated with 1 % SDS showed significant fluorescence of dead cells.

## Electronic supplementary material

Supplementary material 1 (DOCX 166 KB)
