# Supplementary material for: The lipopeptides pseudofactin II and surfactin effectively decrease Candida albicans adhesion and hydrophobicity
Source: Antonie Van Leeuwenhoek. 2015 May 29;108(2):343–53. doi: 10.1007/s10482-015-0486-3 (PMC4491367; doi:10.1007/s10482-015-0486-3)
Supplement: Supplementary file 1 — Supplementary material 1 (DOCX 166 KB) [file 10482_2015_486_MOESM1_ESM.docx]

**Supplementary materials**

‘The lipopeptides pseudofactin II and surfactin effectively decrease *Candida albicans* adhesion and hydrophobicity’

Piotr Biniarz, Gabriela Baranowska, Joanna Feder-Kubis, Anna Krasowska

To test the influence of lipopeptides on the growth of *Candida* strains we monitored the OD at 600 nm of *Candida* cultures containing lipopeptides for 24 hours. Samples were prepared as mentioned before. 96-well microplates were filled with 50 µl of double strength YPG medium and 50 µl of PBS (for control samples) or double strength biosurfactants solutions in PBS. Then, microplates were inoculated with overnight *Candida* cultures to the OD at 600 nm of 0.01. Microplates were incubated for 24 hours at 30 °C. OD_600_ was measured with UMV 340 microplate reader (Asys Hitech, Austria). We detected no influence of tested lipopeptide solutions in the concentrations of 1.5 × CMC on *C. albicans* growth [Fig. A1].


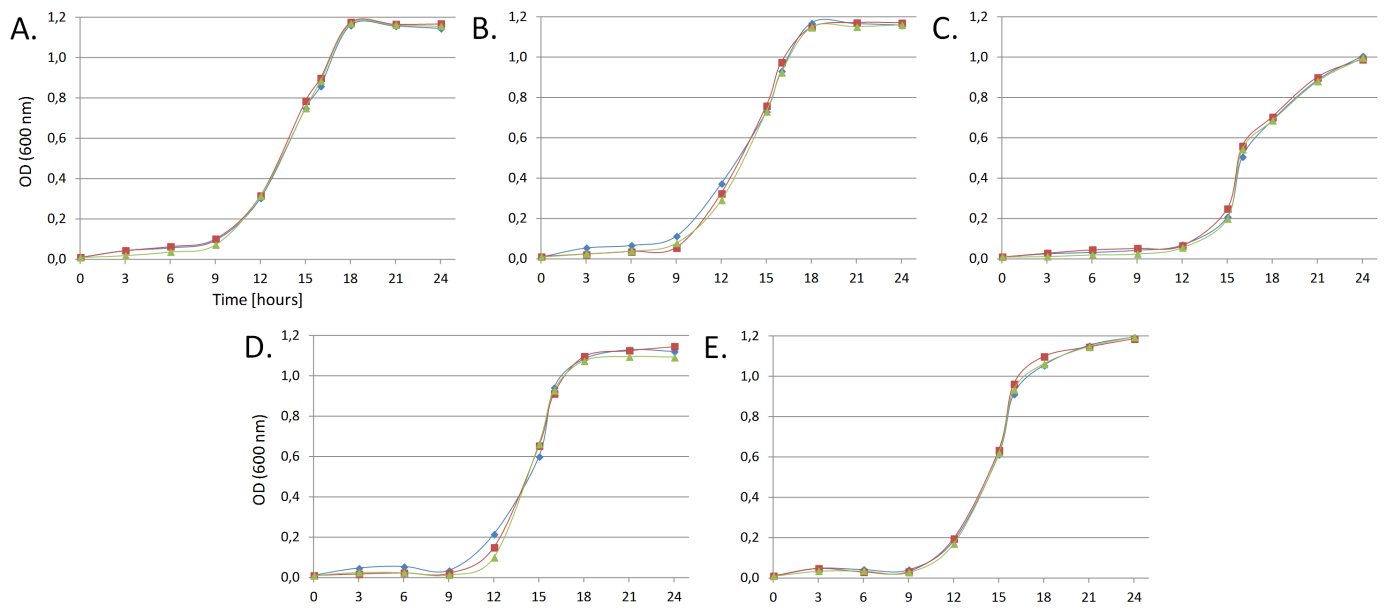


Fig. A1 Growth of *C. albicans* strains SC5315 (A), CAF2-1 (B), CAF4-2 (C), DSY653 (D) and DSY1050 (E) in presence of 0.1 mg/ml PF II (red lines) and 0.015 mg/ml SU (green lines) in comparison to control samples (blue lines). Measurements for each time point were repeated four times.

We also tested if 2-hour preincubation of *Candida* cells with lipopeptides influence their viability by measuring CFU (colony forming units). Briefly, cell suspensions in PBS were transferred to Eppendorf test tubes to reach the final OD_600_ of 0.6. PF II or SU stock solutions in PBS were added to reach the biosurfactant final concentrations (1.5 × CMC). The same amount of PBS was added to the control samples. Suspensions were incubated for 2 hours at 37 ºC with agitation (300 rpm). Then, samples were serially diluted, placed on agar YPG and incubated for 2 days at 30 ºC. Afterwards, grown colonies were counted. Measurements for each sample were done in triplicate and used for calculating means and standard deviations of CFU/ml. We detected no influence of lipopeptides on *C. albicans* viability after 2-hour incubation [Fig. A2].


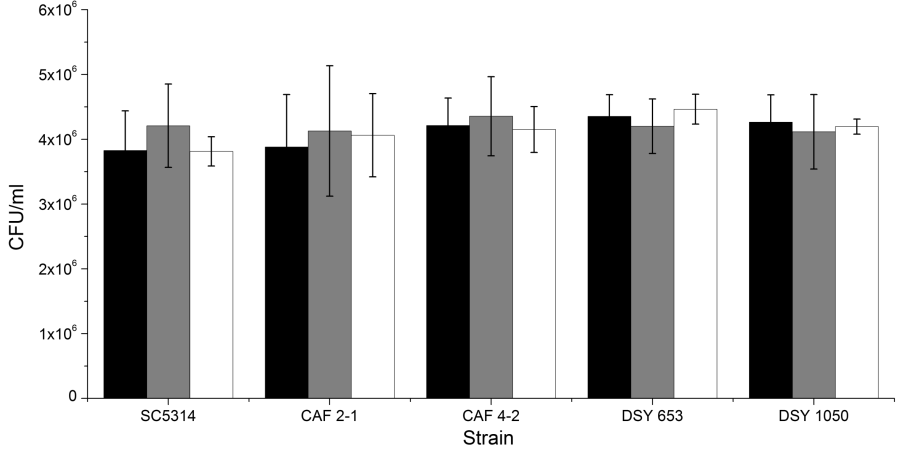


Fig. A2 Viability of *C. albicans* strains after 2-hour preincubation in PBS with 0.1 mg/ml PF II (grey bars) and 0.015 mg/ml SU (white bars) in comparison to control samples (black bars). All measurements were done in triplicate.
